# Toxicity Mechanisms of the Food Contaminant Citrinin: Application of a Quantitative Yeast Model

**DOI:** 10.3390/nu6052077

**Published:** 2014-05-22

**Authors:** Amparo Pascual-Ahuir, Elena Vanacloig-Pedros, Markus Proft

**Affiliations:** 1 Department of Biotechnology, Instituto de Biología Molecular y Celular de Plantas, Universidad Politécnica de Valencia, Ingeniero Fausto Elio s/n, 46022 Valencia, Spain; E-Mail: mevaped@etsia.upv.es; 2 Department of Mechanisms of Plant Stress Responses, Instituto de Biología Molecular y Celular de Plantas, Consejo Superior de Investigaciones Científicas, Ingeniero Fausto Elio s/n, 46022 Valencia, Spain

**Keywords:** citrinin, oxidative stress, yeast, mycotoxins

## Abstract

Mycotoxins are important food contaminants and a serious threat for human nutrition. However, in many cases the mechanisms of toxicity for this diverse group of metabolites are poorly understood. Here we apply live cell gene expression reporters in yeast as a quantitative model to unravel the cellular defense mechanisms in response to the mycotoxin citrinin. We find that citrinin triggers a fast and dose dependent activation of stress responsive promoters such as *GRE2* or *SOD2*. More specifically, oxidative stress responsive pathways via the transcription factors Yap1 and Skn7 are critically implied in the response to citrinin. Additionally, genes in various multidrug resistance transport systems are functionally involved in the resistance to citrinin. Our study identifies the antioxidant defense as a major physiological response in the case of citrinin. In general, our results show that the use of live cell gene expression reporters in yeast are a powerful tool to identify toxicity targets and detoxification mechanisms of a broad range of food contaminants relevant for human nutrition.

## 1. Introduction

Mycotoxins are secondary metabolites produced by many fungi, which represent a major group of hazardous food contaminants [1,2]. Citrinin, (3*R*,4*S*)-8-hydroxy-3,4,5-trimethyl-6-oxo-4,6-dihydro-3*H*-isochromene-7-carboxylic acid, is a well-known mycotoxin, which was first isolated from *Penicillium citrinum*, but can be produced by many other fungal species belonging to the genera *Penicillium*, *Aspergillus* and *Monascus* [3]. Citrinin is an important contaminant of human foods, such as cereals, cheese or sake. It is also found in red yeast rice, widely used in Asia as a food additive or in the elaboration of wine. Citrinin might be even a more common contaminant all over the world since it can be synthesized by the same molds which produce the globally found mycotoxin ochratoxin A. Although the actual mechanism of citrinin toxicity is not entirely understood, in many toxicological models it has been found to cause nephrotoxicity [4]. Several studies in different cell systems seem to confirm that citrinin can generally cause the production of reactive oxygen species (ROS) [5,6,7,8,9]. Moreover, antioxidants have been shown to alleviate the citrinin toxicity [10,11]. Furthermore, several oxidative stress related genes are up-regulated in response to citrinin in yeast according to transcriptomic experiments [12]. However, it is not clear whether this antioxidant defense in yeast is general or restricted to specific enzymatic functions. A recent study from the European Food Safety Authority [13] preliminarily set the maximal citrinin dose of no concern for nephrotoxicity in humans at an exposure level of 0.2 μg/kg body weight per day. For high consuming individuals, especially children, the critical citrinin concentration ranges between 9 and 53 μg/kg grain-based products and for average consumers between 19 and 100 μg/kg grain-based products. However, the same study concluded that the impact of uncertainties on the risk assessment of citrinin is large, and that more data regarding both the occurrence of citrinin in food and feed in Europe and the toxicity mechanisms of this mycotoxin are needed.

Here, we use a yeast (*Saccharomyces cerevisiae*) model to study the toxicology of citrinin. A large body of experimental data confirm that yeast cells respond to many different environmental stresses, including toxins, with the transcriptional activation of so called defense genes in order to survive, adapt and eventually resume growth [14]. The basic idea of the system employed here is that measuring the immediate gene expression response can be indicative of the actual toxicity mechanisms of a particular chemical compound. The use of destabilized luciferase as a reporter allows to quantify stress-induced gene expression in a very sensitive and time resolved manner in living yeast cells [15]. The luciferase assay has the additional advantage that many different environmental conditions, such as different toxin concentrations, *etc*., can be monitored simultaneously. In this way, true dose response patterns are obtained for any toxic compound of interest. The *in vivo* luciferase assay has been very recently applied to decipher dynamic responses of yeast cells to oxidative and saline stress [16]. In the present study we apply specific luciferase reporter fusions to gain insights into the mode of toxicity of citrinin. We find that citrinin triggers an immediate response to oxidative stress characterized by a strong and dose-dependent induction of natural genes. Furthermore, the oxidative stress responsive transcription factors Yap1 and Skn7 are critically involved in the adaptive gene expression triggered by citrinin. More specifically, Yap1 dependent artificial luciferase reporters are highly responsive to citrinin. Genetic manipulations which eliminate specific multidrug export systems of yeast increase the citrinin toxicity. Altogether the results presented here strongly suggest that oxidative damage might be the prevalent and immediate toxicity mechanism of the mycotoxin citrinin.

## 2. Materials and Methods

### 2.1. Yeast Strains and Growth Conditions

*Saccharomyces cerevisiae* strains used in this study were: wild type BY4741 (*MATa*; *his3Δ1*; *leu2Δ0*; *met15Δ0*; *ura3Δ0*) and the mutant alleles *yap1::KanMX4*; *skn7::KanMX4*; *pdr1::KanMX4*; *pdr5::KanMX4*; *snq2::KanMX4*. For luciferase assays the cells were transformed with the respective lucCP^+^ fusion plasmids and grown in Synthetic Dextrose (SD) medium which contained 0.67% Yeast Nitrogen Base, 50mM succinic acid pH 5.5, 2% dextrose, 100 mg/L methionine, 100 mg/L leucine and 25 mg/L uracil. For citrinin sensitivity assays on agar plates, the respective yeast strains were grown in yeast extract-peptone liquid medium containing 2% dextrose (YPD) to exponential growth phase and then incubated with the indicated concentrations of citrinin.

### 2.2. Plasmid Constructions

The destabilized luciferase reporter fusions with the natural *GRE2* or *SOD2* promoter are described elsewhere [15,16]. Briefly, the *GRE2*-lucCP^+^ fusion contains the upstream 940 nucleotides of the *GRE2* gene fused with the destabilized luciferase lucCP^+^ gene in a centromeric *HIS3* containing yeast expression plasmid. The *SOD2*-lucCP^+^ fusion contains the upstream 977 nucleotides of the *SOD2* gene in the same vector backbone. The destabilized luciferase reporters with the specific promoter elements STRE, CRE or AP-1 are described in [16]. Briefly, they contain triple insertions of each cis-element in the *CYC1* core promoter fused to lucCP^+^ in centromeric *HIS3* containing yeast expression plasmids. 

### 2.3. Live Cell Luciferase Assays

Yeast strains transformed with the respective luciferase reporter plasmids were grown at 28 °C overnight in SD medium to OD = 2 at 600 nm. The culture volume necessary for the entire luciferase assay was incubated on a roller at 28 °C for 90 min with 0.5 mM luciferin (Sigma, St. Louis, MO, USA) from a 10 mM stock solution in DMSO. The culture was then distributed in 120 μL aliquots in white 96-well plates (Nunc, Penfield, NY, USA) and the indicated concentrations of citrinin were added from a stock solution in DMSO. The mock treated samples contained the same concentration of solvent without the mycotoxin. The light emission from the culture aliquots was continuously recorded in a GloMax Multidetection System (Promega, Madison, WI, USA) in the luminometer mode. Data were normalized for the absolute number of cells used in the assay and processed in Microsoft Excel. For each condition, three independent culture aliquots were analyzed.

### 2.4. Yeast Sensitivity Assays

For plate assays, the yeast strains under study were grown in YPD liquid medium to exponential growth phase. Culture aliquots were then distributed in multiwell plates and exposed for 3 h to the indicated concentrations of citrinin added from a stock solution in DMSO. Equal amounts of cells were then plated on fresh YPD agar plates, which were incubated at 28 °C for 2 days.

For the Fluorescein Diacetate (FDA) quantification of live cells after acute citrinin exposure, the respective yeast strains were pregrown in liquid YPD medium to exponential growth phase. The amount of cells necessary for the complete assay was washed and resuspended in potassium phosphate (KP) buffer (KH_2_PO_4_/K_2_HPO_4_ pH 7.4). The indicated doses of citrinin were applied to 120 μL cell aliquots in black 96 well plates (Nunc) for 1 h. FDA (Sigma) was added from a 5 mg/mL stock solution in acetone to a final concentration of 250 μg/mL. After 10 min the fluorescence was quantified in a GloMax Multidetection System (Promega) in the fluorescence mode with excitation at 490 nm and emission at 510–570 nm. Three independent culture aliquots were measured for each condition. Fluorescence levels were corrected by subtracting the fluorescence produced by the same amount of dead cells, which were obtained by incubation at 80 °C for 60 min.

### 2.5. Statistics

The luciferase reporter assays were performed with three independent yeast culture aliquots. The standard deviation (SD) in this assay is <15%; however, error bars are not indicated in the graphs to make the figures clearly visible. For citrinin sensitivity assays, three independent culture aliquots were analyzed. The significance of the differences in the viability among different mutant strains was assessed by the application of the Student’s *t*-test.

## 3. Results

**Figure 1:**
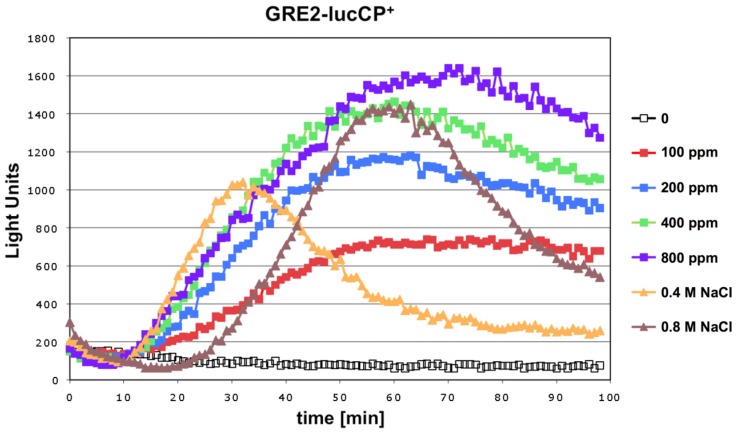
Citrinin activates gene expression from stress responsive yeast promoters in a dose dependent fashion. A fusion of the stress inducible *GRE2* promoter with destabilized luciferase was used as a real time reporter for gene expression. Citrinin doses from 100–800 ppm were applied to the yeast cultures. Alternatively the *GRE2*-luciferase reporter was activated by the indicated concentrations of NaCl. Data shown are mean values from three independent biological samples. SD < 15%.

We tested whether exposure to the mycotoxin citrinin caused a rapid adaptation of gene expression in yeast. An immediate and transient activation of defense gene expression is a common adaptive response of this organism to a great variety of environmental threats or stresses. Live cell reporters based on the expression of a destabilized luciferase enzyme represent a sensitive method to monitor this adaptive response in real time and upon a gradual range of stress conditions. We first examined a reporter based on a natural yeast promoter (*GRE2*), which is responsive to different types of stress such as hyperosmotic stress or oxidative stress [17]. As shown in Figure 1, the *GRE2*-luciferase reporter is readily activated upon acute exposure to citrinin. Robust reporter activation was observed for citrinin doses equal or greater than 100 ppm. Importantly the citrinin response of *GRE2* was comparable to the activation of the same reporter by salt stress (Figure 1), which is known to trigger a very strong increase of *GRE2* expression [16,18]. A dose dependent increase in the luciferase reporter activity was observed in a citrinin concentration range from 100 to 400 ppm. Further increases in the toxin dose did not yield a significantly enhanced gene expression response. These results made us confident that the citrinin response of the *GRE2*-luciferase reporter reflected a biologically meaningful adaptation of yeast to its toxicity, which was further investigated with refined reporter systems.

**Figure 2:**
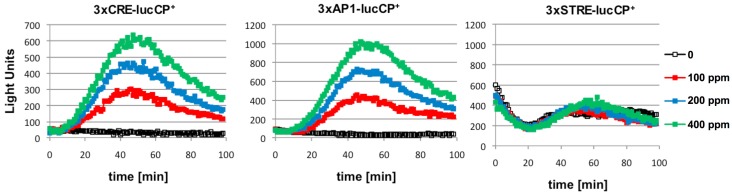
Citrinin activates gene expression from CRE and AP-1 promoter elements in a dose dependent fashion. Artificial promoter-luciferase constructs were used, which contained multiple repetitions of the same cis-element: CRE, AP-1 or STRE as indicated. The indicated citrinin doses were applied at time point 0 to the yeast cultures and the light emission continuously monitored. Data shown are mean values from three independent biological samples. SD < 15%.

We next used yeast wild type cells, which were transformed with more specific luciferase reporters, for further citrinin studies. The insertion of multiple copies of just one type of transcription factor binding motif into luciferase expression plasmids, has been shown to create very specific reporters which respond to stimuli via just one or few signal transduction pathways [15,16]. Here, we investigated three types of cis elements: STRE (bound by the Msn2/4 factors in response to general stress), CRE (bound by the Sko1 factor in response to osmotic stress and by unknown factors in response to oxidative stress), AP-1 (bound by Yap1 in response to oxidative stress) [16]. As depicted in Figure 2, we found that citrinin exposure activated gene expression from AP-1 and CRE sites, but not from STRE elements. Since yeast AP-1 promoter elements are exclusively activated by oxidative damage, this was a clear indication that citrinin provoked intracellular oxidation, which then activated adaptive gene expression via oxidative stress responsive transcription factors such as Yap1. We further confirmed this by showing that activation of AP1-driven luciferase expression by citrinin was completely absent in a *yap1* mutant strain (data not shown).

**Figure 3:**
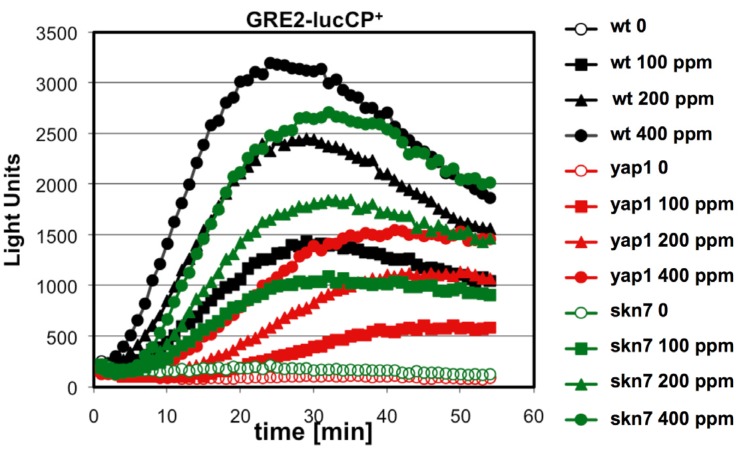
Citrinin activates gene expression via the oxidative stress responsive transcription factors Yap1 and Skn7. A fusion of the stress inducible *GRE2* promoter with destabilized luciferase was used as a real time reporter for gene expression. The reporter activity was measured in yeast wild type and *yap1* or *skn7* mutants upon addition of the indicated concentrations of citrinin. Data shown are mean values from three independent biological samples. SD < 15%.

To further investigate the relation between citrinin toxicity and oxidative stress signaling, we monitored the toxin induced gene expression in yeast strains which lacked the activity of the two main transcriptional activators operating upon oxidative stress: Yap1 and Skn7. We used the *GRE2*-luciferase reporter system to compare the immediate transcriptional upregulation upon citrinin exposure in wild type compared to *yap1* or *skn7* knockout mutants. As shown in Figure 3, in the absence of Yap1 the citrinin induced reporter activity was severely reduced. Yap1 might therefore be one of the main transcriptional regulators which is activated in response to citrinin stress. Skn7 seemed to be additionally involved in the citrinin response; however, its contribution was clearly less important as compared to Yap1 (Figure 3).

**Figure 4:**
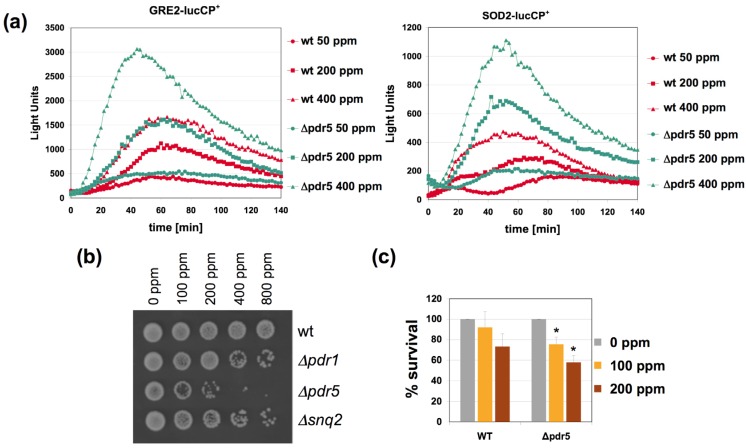
The yeast Pdr5 multidrug transporter is important for the citrinin dose response and sensitivity. (**a**) Fusions of the stress inducible *GRE2* (**left panel**) or *SOD2* (**right panel**) promoters with destabilized luciferase were used as a real time reporter for gene expression. The reporter activity was measured in yeast wild type and *pdr5* mutants upon addition of the indicated concentrations of citrinin. Data shown are mean values from three independent biological samples. SD < 15%; (**b**) *pdr5* mutants show increased sensitivity to citrinin. The indicated citrinin doses were applied to yeast wild type, *pdr1*, *pdr5* and *snq2* mutants for three hours in YPD culture medium. Surviving cells were then assayed on a fresh YPD plate; (**c**) Yeast wild type and *pdr5* mutant cells were incubated for one hour with the indicated amounts of citrinin in KP buffer. The amount of living cells was then quantified by staining with FDA. Data are mean values from three independent biological replicas. Error bars are SD. The asterisks refer to *p* < 0.05 different from wt in the same condition according to the Student’s *t*-test. The value for mock treated cells was arbitrarily set to 100 for both yeast strains.

We next wanted to gain insights into the molecular mechanisms of citrinin extrusion and its effect on its toxicitiy. The yeast genome encodes a large family of multidrug transporters which are pleiotropically involved in the export of many xenobiotic chemicals from the cytosol. Two genes of this transporter family, *PDR5* and *SNQ2*, have been identified in yeast as up-regulated upon citrinin exposure [12]. Additionally we included a yeast strain, *Δpdr1*, in the citrinin study, which lacks one of the principal transcriptional activators of the multidrug resistance gene family [19]. As shown in Figure 4, the *pdr5* mutant strain showed the highest degree of sensitivity to citrinin in a growth assay in rich medium (Figure 4B). The enhanced toxicity of citrinin in a *pdr5* mutant was further confirmed by an independent assay using FDA as a live cell stain upon acute citrinin stress in potassium phosphate buffer (Figure 4C). These results indicated that Pdr5 was a major citrinin export activity in yeast and we postulated that *pdr5* mutant cells were more sensitive to the mycotoxin because of greater accumulation of citrinin in the cell interior. We therefore measured the adaptive response to citrinin in *pdr5* mutants and compared it to wild type. As shown in Figure 4A, the dose dependent response of two citrinin inducible luciferase reporters, *GRE2* and *SOD2*, was largely enhanced in the *pdr5* mutant strain. Therefore we can confirm that the lack of the Pdr5 multidrug transporter leads to hypersensitivity to citrinin and a more sensitive adaptive response to the toxin, presumably caused by overaccumulation of citrinin inside the cell.

## 4. Discussion

Citrinin is an important food contaminant produced by *Penicillium*, *Aspergillus* and *Monascus* species. Although it is generally classified as a nephrotoxic compound, its principle mechanisms of toxicity are not entirely clear. Various *in vitro* studies have determined different possible pathways and sources of cellular damage including lipid peroxidation, mitochondrial dysfunction or the induction of apoptotic cell death [5,20,21,22]. Additionally, genotoxic effects of citrinin have been confirmed by the enhanced formation of micronuclei in different animal and human cell lines [23,24,25,26]. However, the primary mechanisms of citrinin toxicity remain elusive. In the present study we use a yeast based reporter system to gain insights into the immediate cellular response to the exposure to citrinin. Yeast cells are an excellent cellular model to study the adaptive response to diverse chemical threats, since these unicellular organisms have evolved efficient detoxification paths, which assure cell survival and are often triggered by rapid transcriptional activation of defense systems. Moreover, the application of live cell reporter assays enables to monitor the dose sensitive stress response in a small aliquot of living yeast cells in real time [15,16]. Application of this monitoring method clearly shows that citrinin triggers an immediate adaptive response related to the oxidative stress defense. Citrinin provokes the rapid up-regulation of oxidative stress responsive genes such as *GRE2* or *SOD2*. Of note, the mitochondrial enzyme superoxide dismutase encoded by *SOD2* has a well defined antioxidant function upon sudden bursts of reactive oxygen species (ROS) [27]. Therefore, one of the primary and immediate toxic effects of citrinin is very likely the generation of high ROS levels, which are targeted by the yeast cell by the activation of enzymatic antioxidants. Several lines of evidence indicate that citrinin induced ROS levels must be a critical determinant of the toxicity of this mycotoxin: a- citrinin induces the expression of the *GRE2* or *SOD2* genes to levels which are comparable to their most potent natural inducers such as NaCl or hydrogen peroxide [16]; b- transcriptional regulators specifically involved in the yeast antioxidant response, such as Yap1 or Skn7, are important for the efficient activation of gene expression upon citrinin treatment; c- the dose sensitive transcriptional activation triggered by citrinin (50–400 ppm; 0.2–1.6 mM) occurs in a similar concentration range as compared to hydrogen peroxide (0.1–1.0 mM) [16].

Our data are in agreement with transcriptomic surveys performed in yeast upon citrinin stress, which identified some antioxidant functions to be up-regulated in response to the mycotoxin [12]. Iwahashi and coworkers identified a limited number of genes with a confirmed or presumed function in the oxidative stress response including some *AAD* genes (hypothetical aryl-alcohol dehydrogenases), *OYE3* (NADPH oxidoreductase), *GRE2* (methylglyoxal reductase), and *TRX2* (thioredoxin) [12]. However, it was important to prove whether citrinin triggers a general antioxidant response in cells. This is confirmed by our study here by the use of oxidative stress specific reporter genes. The most direct proof that citrinin primarily causes intracellular oxidation is the robust activation of a reporter gene controlled by the AP-1 promoter element, which is known to be selectively and exclusively activated by increases in intracellular ROS via the Yap1 transcription factor [16,28,29]. Of note, citrinin causes an immediate activation of oxidative stress specific reporter genes very similar to well known and potent pro-oxidants such as hydrogen peroxide or menadione [8]. Additionally the antioxidant response is observed here in glucose containing growth medium, which is a fermentative condition repressing mitochondrial energy metabolism in yeast. These facts indicate that citrinin directly damages cellular components different from mitochondria. Our data obtained in the yeast model confirm previous reports in higher cell lines demonstrating that citrinin is able to trigger oxidative stress [5,6,7,8,9]. This is important because a possible antioxidant function has been proposed for citrinin, which does not seem to be relevant *in vivo* and might be attributable to specific chemical derivatives of this mycotoxin [30].

Yeast cells seem to be inherently more resistant to citrinin as compared to higher eukaryotic cell lines. In our hands, a yeast wild type begins to mount a measurable gene expression response at citrinin doses around 50 ppm (200 μM) and easily survives treatments as high as 200 ppm (800 μM). Mammalian cell cultures show significant genotoxic damage and loss of viability already at citrinin concentrations of approximately 50 μM [24]. An important part of the observed citrinin tolerance is likely the efficient extrusion of the toxin from the interior of the yeast cell. Iwahashi and coworkers identified two multidrug resistance transporter encoding genes to be up-regulated upon citrinin exposure [12]. According to our results, one of them (Pdr5), is especially important for citrinin tolerance. Yeast cells lacking Pdr5 are hypersensitive to citrinin and trigger a greater adaptive response to the toxin presumably because this strain accumulates citrinin to higher intracellular levels. An additional barrier for citrinin toxicity might also be the yeast cell wall. The outer envelope together with efficient efflux systems might therefore make yeast cells more resistant to citrinin and shift their transcriptional response to doses which are higher than normally found in contaminated food. Taken together, our study demonstrates that yeast serves as an efficient model to unravel toxicity mechanisms and detoxification strategies upon exposure to human food contaminants such as mycotoxins.

## 5. Conclusions

The mycotoxin citrinin triggers an immediate and general antioxidant response in yeast cells. Induction of harmful ROS levels might therefore be the prevalent toxicity mechanism of this toxin. In yeast cells, citrinin activates the expression of antioxidant encoding genes and oxidative stress specific reporters. The ROS activated transcription factor Yap1 is critically involved in the adaptive response to citrinin. Additionally, the mutation of specific toxin exporters such as Pdr5, identifies physologically important citrinin defense systems. Yeast is an efficient model to unravel toxicity and detoxification mechanisms of mycotoxins.
